# Glomus tumor mimicking digital adenocarcinoma

**DOI:** 10.1093/jscr/rjad307

**Published:** 2023-05-30

**Authors:** Noelani-Mei Ascio, Dimitri Tchienga, Abid Qureshi, David Liu, Armand Asarian, Romulo Genato, Philip Xiao

**Affiliations:** School of Medicine, St. George’s University, Grenada, WI, USA; School of Medicine, St. George’s University, Grenada, WI, USA; Department of Surgery, The Brooklyn Hospital Center, Brooklyn, NY, USA; Department of Pathology, The Brooklyn Hospital Center, Brooklyn, NY, USA; Department of Surgery, The Brooklyn Hospital Center, Brooklyn, NY, USA; Department of Surgery, The Brooklyn Hospital Center, Brooklyn, NY, USA; Department of Pathology, The Brooklyn Hospital Center, Brooklyn, NY, USA

**Keywords:** Glomus, Soft tissue tumors, Digital adenocarcinoma, Smooth muscle actin, Immunohistochemistry stain

## Abstract

Glomus tumors are uncommon, benign lesions commonly located on the digits of the hands and are diagnostically challenging. This is because hemangiomas or ganglion cysts are more commonly identified in those locations. Our case report underlines the diagnostic challenge of a glomus tumor and the importance of immunohistochemical staining.

## INTRODUCTION

Glomus tumors are relatively uncommon benign tumors that typically appear in the subungual region of the digits. They originate from glomus bodies, which are arteriovenous anastomoses deep to the epidermis, which help to regulate blood flow to the extremities [1]. Although glomus tumors can be mistaken for the other more common types of benign digital lesions like lipomas or cysts, they should not be mistaken for digital adenocarcinoma (DA), an even rarer type of tumor with high metastatic potential [2, 3]. Therefore, DA should be considered in the differential diagnoses when there is suspicion of lesions with the appearance of a glomus tumor. This report describes the diagnostically challenging case of a pathologically suspected DA on morphology in a patient then found to have glomus tumor by immunohistochemical stain.

## CASE PRESENTATION

Our patient is an 62 years old male with a past medical history of hypertension, hyperlipidemia, diabetes mellitus and hyperthyroidism, and he presented with a 5 mm tender, soft subungual mass on his left index finger. He had a nail abnormality with distraction of the nail running from the region of the mass distally. He subsequently underwent excision of the mass and there were no postoperative complications.

## PATHOLOGY FINDINGS

**Figure 1:**
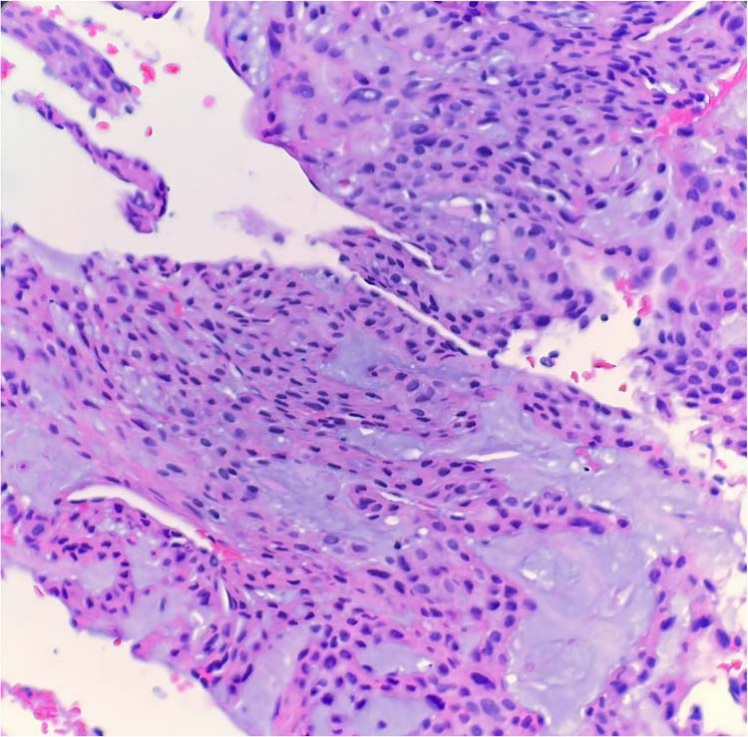
Microscopic examination reveals epithelioid bland tumor cells forming glandular structure with mucinous material. H&E ×40.

**Figure 2:**
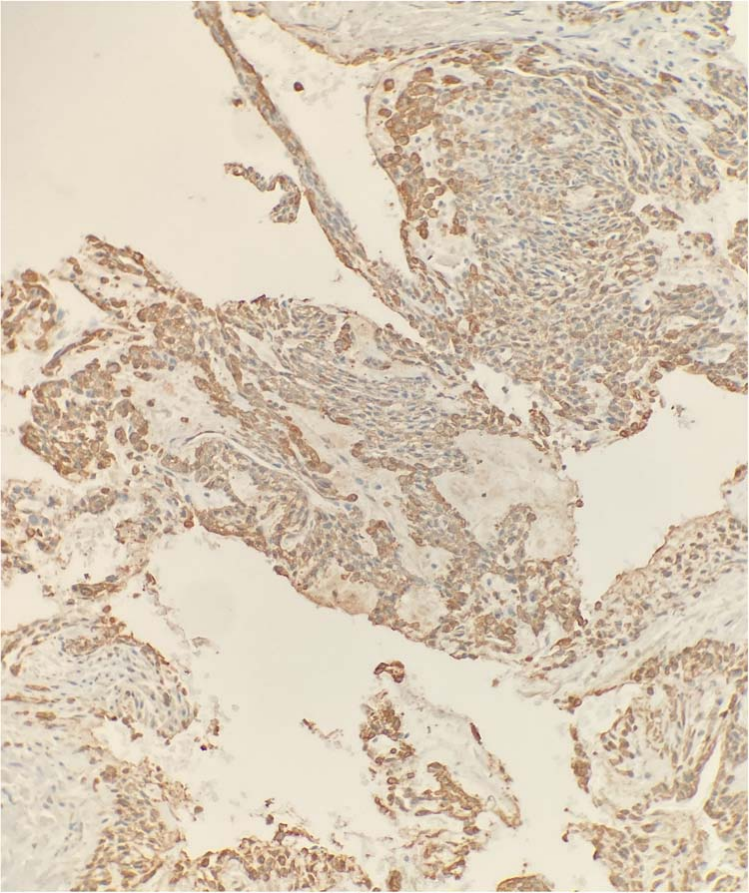
Immunohistochemical stain reveals that tumor cells are positive for SMA. IHC ×20.

The excised mass was a fragment of tan red soft tissue measuring 0.7 × 0.4 × 0.4 cm. Microscopic examination reveals that tumor is composed of epithelioid bland cells forming gland-like architecture with scant mucinous material (Fig. 1). Without immunohistochemical stain, the morphological features are diagnostic for adenocarcinoma. However, in order to rule out other diagnostic possibility, we performed smooth muscle actin (SMA) immunohistochemical stain. The tumor cells turn out to be positive for SMA (Fig. 2). Combined with this immunoprofile, the final diagnosis of glomus tumor was rendered. The diagnosis of glomus tumor was confirmed with second opinion at world renowned institute.

## DISCUSSION

Glomus tumors account for ~1.6% of soft tissue tumors [4]. They commonly affect both genders, but subungual lesions are more prevalent in females, with a ratio of 3:1 [1]. Patients with glomus tumors, which arise from the glomus body, often complain of pain, especially in response to cold temperatures [1]. Glomus tumors can be divided into benign, uncertain malignant potential and malignant. A glomus tumor of uncertain malignant potential is considered when the tumor is deep (<2 cm) with atypical mitotic figures. Malignant glomus tumors are characterized by marked nuclear atypia, mitotic activity and atypical mitotic figures [1]. In contrast, features of DA on histopathological examination are ‘basaloid/cuboidal to low columnar epithelial cells and myoepithelial cells with mild to moderate cytologic atypia’ [4].

The presence of epithelioid cells in both glomus tumor and adenocarcinoma may explain the similarity in our report. In addition to epithelioid cells [5], glomus tumors were reported mimicking adenocarcinoma [6], gastrointestinal stromal tumor [7] and urothelial carcinoma [8]. It has been hypothesized that glomus tumors are mesodermal derivatives but not true neoplasm. Their origin may result from an increase in vasculature accompanied by the mural cells changing into proliferating glomus cells [5].

Careful evaluation of the pathological findings is essential to distinguish between glomus tumors and adenocarcinoma. SMA is a specific marker with 99% specificity for the diagnosis of glomus tumor [2]. SMA is commonly used to identify myoepithelial cells, smooth muscle cells and myofibroblasts in normal, reactive or neoplastic tissue, which should not be seen in adenocarcinoma [9]. In our report, the initial evaluation on morphology suggested adenocarcinoma because of glandular like structures. Because of the positive of SMA, the final diagnosis of glomus tumor was made. Further studies including immunohistochemical stain are necessary to fully understand the histological similarities and dissimilarities between the two types of tumors, in order to differentiate the benign lesions from malignant neoplasms.

## CONCLUSION

Glomus tumors can mimic other types of tumors, including adenocarcinoma, and can present a diagnostic challenge. Careful evaluation of the pathological findings, including the use of immunohistochemical markers, is necessary to accurately diagnose these tumors.

## CONFLICT OF INTEREST STATEMENT

None declared.

## FUNDING

None.
